# Targeting Functionally Characterized Synaptic Architecture Using Inherent Fiducials and 3D Correlative Microscopy

**DOI:** 10.1017/S1431927620024757

**Published:** 2021-02

**Authors:** Connon I. Thomas, Melissa A. Ryan, Benjamin Scholl, Debbie Guerrero-Given, David Fitzpatrick, Naomi Kamasawa

**Affiliations:** 1 Electron Microscopy Core Facility, Imaging Center, Max Planck Florida Institute for Neuroscience, 1 Max Planck Way, Jupiter, FL 33458, USA; 2 Functional Architecture and Development of Cerebral Cortex, Max Planck Florida Institute for Neuroscience, 1 Max Planck Way, Jupiter, FL 33458, USA

**Keywords:** 3D CLEM, EM-label-free, *in vivo* 2-photon microscopy, neuronal circuits, SBF-SEM

## Abstract

Brain circuits are highly interconnected three-dimensional structures fabricated from components ranging vastly in size; from cell bodies to individual synapses. While neuronal activity can be visualized with advanced light microscopy (LM) techniques, the resolution of electron microscopy (EM) is critical for identifying synaptic connections between neurons. Here, we combine these two techniques, affording the advantage of each and allowing for measurements to be made of the same neural features across imaging platforms. We established an EM-label-free workflow utilizing inherent structural features to correlate *in vivo* two-photon LM and volumetric scanning EM (SEM) in the ferret visual cortex. By optimizing the volume SEM sample preparation protocol, imaging with the OnPoint detector, and utilizing the focal charge compensation device during serial block-face imaging, we achieved sufficient resolution and signal-to-noise ratio to analyze synaptic ultrastructure for hundreds of synapses within sample volumes. Our novel workflow provides a reliable method for quantitatively characterizing synaptic ultrastructure in functionally imaged neurons, providing new insights into neuronal circuit organization.

## Graphical Abstract

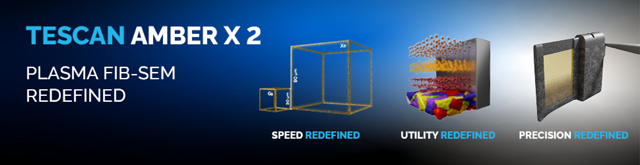

## Introduction

To understand how the brain processes and encodes information, neuronal networks must be investigated both functionally and anatomically. In light microscopy (LM), the development of activity-dependent fluorophores, such as GCaMP (Nakai et al., 2001), and improvements to depth and resolution of live imaging techniques enable quantification of neuronal function at the level of individual dendritic spines, a major site of cell-to-cell communication (Ji et al., 2016; Lu et al., 2017; Scholl et al., 2017; Luo et al., 2018; Moyer & Zuo, 2018). While LM methods can capture dynamic activity in neural compartments, they are unable to resolve fine synapse morphology because the size of individual synapses and their components are below the light diffraction limit. Electron microscopy (EM), therefore, remains the “gold-standard” for accessing features of synaptic ultrastructure.

Combining distinct imaging modalities has often proved difficult; to date, simultaneous measurement of structure and function in the same cell has only been achieved for a few specifically integrated LM-EM microscopic techniques (Agronskaia et al., 2008; Peddie et al., 2014; de Boer et al., 2015; Timmermans & Otto, 2015; Koning et al., 2019; Mohammadian et al., 2020). Because of this, correlative light and electron microscopy (CLEM) workflows beginning with LM and ending with EM are routine, allowing for sequential observation of volumes of tissues [reviewed in Collinson et al. (2017), Collinson and Verkade (2019)]. Early CLEM workflows for neuroscience were developed with cultured neurons and relied heavily on external fiducial markers such as indexed grids or carbon-marker coated glass for growing cells, techniques which are incompatible with intact tissue. Infiltration of nano-gold particles, quantum dots, or nano-diamonds into cells has also been used (Giepmans, 2008; Weston et al., 2009; de Boer et al., 2015), but these methods damage cellular structures (Weston et al., 2009; Polishchuk & Polishchuk, 2019).

More recently, CLEM workflows are enabling consecutive functional and structural imaging of the same tissue across several modalities using genetically encoded markers, near-infrared branding, or inherent fiducials for feature re-identification (Bishop et al., 2011; Maco et al., 2013; Ellisman et al., 2015; Collinson et al., 2017; Goetz, 2018; Lippens & Jokitalo, 2019). Peroxidases such as HRP, APEX2, and miniSOG can be genetically expressed selectively within cells and/or organelles and are used to target cells with minimal damage to structures; however, these labeling techniques can still obscure or disrupt ultrastructural features when too much reaction product is produced (Shu et al., 2011; Ellisman et al., 2012; Horstmann et al., 2013; Lam et al., 2015; Martell et al., 2017; Shigemoto & Joesch, 2017; Thomas et al., 2019). To limit potential disruption of the sample, use of biological fiducials such as cell somata, blood vessels, and other intrinsic structures as correlative markers has been successful in several different organisms and brain regions, providing a method of correlation without labeling issues (Bock et al., 2011; Goetz et al., 2014; Karreman et al., 2016; Lee et al., 2016; Drawitsch et al., 2018; Luckner et al., 2018).

As the scale of *in vivo* LM technology has massively increased, allowing collection of large tissue volumes or simultaneously targeting a larger number of features (Lu et al., 2017; Forli et al., 2018; Yang et al., 2018), the need for paired volumetric ultrastructural EM data of targeted neuronal features has grown in turn. To achieve this goal, three-dimensional (3D) CLEM utilizing automated imaging has paved the way to collecting large volumes of EM data more efficiently (Karreman et al., 2016; Collinson et al., 2017; Kubota et al., 2018; Lippens & Jokitalo, 2019). Serial block-face SEM (SBF-SEM) is one such technique that uses a diamond knife within the microscope to serially section and image tissues automatically (Denk & Horstmann, 2004). However, SBF-SEM also presents several challenges involving sectioning and imaging thick biological samples *in situ*, especially when acquiring high-resolution images. Tissues require extensive heavy metal staining to increase conductivity and reduce charging artifacts produced by the electron beam. Achieving even and well-contrasted staining of lipids and proteins throughout the tissue can sometimes require protocol modification depending on the thickness and type of tissue (Tapia et al., 2012; Hua et al., 2015; Mikula & Denk, 2015; Genoud et al., 2018). Obtaining a sufficient signal-to-noise ratio (SNR) in samples serially imaged using standard backscattered-electron detectors requires long beam dwell times, often damaging the sample and extending imaging times. Finally, correlation of small features within large volumes remains technically challenging as the brain tissue is incredibly dense and uniform.

Here, we describe a 3D-CLEM workflow designed to re-identify dendritic spines on neurons in ferret visual cortex that have been imaged *in vivo* under 2-photon LM (to assess function) within volumes collected with SBF-SEM (to measure ultrastructure). Correlation of features among 2-photon LM, confocal laser-scanning microscopy (CLSM) and SBF-SEM required: (1) modification of the EM sample preparation protocol to visualize the fine morphology of synapses in EM while creating sufficient membrane-cytoplasm contrast; (2) development of a reliable and reproducible feature relocation method using inherent fiducials; (3) optimizing image size, SNR, and resolution while minimizing charging and imaging time; and (4) utilizing virtual reality as a technique for image and volume correlation.

## Materials and Methods

All procedures were performed according to NIH guidelines and were approved by the Institutional Animal Care and Use Committee at the Max Planck Florida Institute for Neuroscience.

### Animals

Viral injections, anesthesia, cranial window preparation, and presentation of visual stimuli for behavioral experimentation in ferrets are described in depth in our previous works (Scholl et al., 2017, 2020). Briefly, layer 2/3 neurons of the primary visual cortex of female ferrets (*n* = 3) were driven to express the calcium indicator GCaMP6s via a Cre-dependent viral expression system. A cranial window was surgically implanted to image calcium activity of cortical pyramidal neurons during presentation of visual stimuli, in the form of drifting gratings. Change in fluorescence (Δ*F*/*F*_0_) of the soma and dendritic spines was recorded using two-photon microscopy as a measure of cell and spine activity.

### Two-Photon Imaging

Two-photon imaging was performed using a Bergamo II microscope (Thorlabs) running Scanimage (ver. 5, Vidrio Technologies) with 940-nm dispersion-compensated excitation provided by an Insight DS+ (Spectraphysics). For spine and axon imaging, power after the objective was limited to <50 mW. Images were collected at 30 Hz using bidirectional scanning with 512 × 512 pixel resolution or with custom ROIs (region of interests; frame rate range: 22–50 Hz). Somatic imaging was performed with a resolution of 0.488–0.098 *μ*m/pixel. Dendritic spine imaging was performed with a resolution of 0.164–0.065 *μ*m/pixel.

### Perfusion, Fixation, and Slice Preparation for Fluorescence Imaging

Anesthetized animals were immediately perfused with 2% paraformaldehyde and 2–2.5% glutaraldehyde in a 0.1 M sodium cacodylate buffer (pH 7.4). Following removal, brains were sliced at 80 *μ*m parallel to the area flattened by the cranial window. Slices were quickly imaged at low magnification (20×, 0.848 × 0.848 *μ*m/pixel) using a Leica CLSM TCS SP5 II running LAS AF (ver. 3.0, Leica) with 488 nm laser excitation. Fluorescence of GCaMP6s was used to locate the target cell in this view. Autofluorescence resulting from glutaraldehyde fixation also produced signal within the tissue slices. A field of view large enough to cover roughly one-fourth of the slice, with the target cell included, was captured using image tiling. This process was performed to identify the fluorescent cell of interest and for slice-level correlation in later steps of the workflow. The slice containing the target cell was then imaged with the same 20× objective at higher pixel resolution (0.360 × 0.360 × 0.976 *μ*m/voxel) to obtain a z-stack of the full depth of the slice and immediate region surrounding the target cell. CLSM imaging required approximately 1–3 h to find the cell of interest within a slice and capture a tiled slice overview and higher resolution z-stack.

### Sample Preparation for SBF-SEM Imaging

For CLEM imaging of ferret cortex, we tested multiple modifications of the original protocols for 3D-volume EM established by Deerinck et al. (2010) with the goal of developing a protocol that achieved uniform staining, high conductivity, and excellent cytoplasm/membrane contrast. The details of all protocols tested are displayed in Table 1. To evaluate the effects of each protocol, we cut ultrathin sections with 50 nm thickness (UC7, Leica) and examined them using TEM at 100 kV (Tecnai Spirit, Thermo Fisher Scientific) without counterstaining. The final protocol that provided optimal imaging conditions which we used for data collection is described here.

Following LM imaging and before EM sample preparation, slices were imaged under a dissecting microscope and the ROI was identified by correlating blood vessel to those visualized by CLSM using manual overlay in Photoshop (CS6 ver. 13.0.1, Adobe). The slice correlation step required a few minutes. Then, slices were trimmed to less than 2 × 2 mm with the target cell at the center (see Fig. 3 for details). Tissue pieces were incubated in an aqueous solution of 2% osmium tetroxide buffered in 0.1 M sodium cacodylate for 45 min at room temperature (RT). Tissue was not rinsed and the osmium solution was replaced with cacodylate buffered 2.5% potassium ferrocyanide for 45 min at RT in the dark. Tissue was rinsed with water 2 × 10 min, which was repeated between consecutive steps. Tissue was incubated at room temperature for 20 min in aqueous 1% thiocarbohydrizide dissolved at 60°C, aqueous 1% osmium tetroxide for 45 min at RT, and then 1% uranyl acetate in 25% ethanol for 20 min at RT in the dark. Tissue was rinsed then left in water overnight at 4°C. The following day, tissue was stained with Walton’s lead aspartate for 30 min at 60°C. Tissue was then dehydrated in a graded ethanol series (30, 50, 70, 90, and 100%), 1:1 ethanol to acetone, then 100% dry acetone. Tissue was infiltrated using 3:1 acetone to Durcupan resin (Sigma-Aldrich) for 2 h, 1:1 acetone to resin for 2 h, and 1:3 acetone to resin overnight, then flat embedded in 100% resin on a glass slide and covered with an Aclar sheet at 60°C for 2 days. The tissue was trimmed to less than 1 × 1 mm in size, the empty resin at the surface was shaved to expose the tissue surface using an ultramicrotome (UC7, Leica), then turned downwards to be remounted to a metal pin with conductive silver epoxy (CircuitWorks, Chemtronics).

For the 3View detector test, we used one adult Wistar rat brain formerly fixed and prepared for the SBF-SEM. The animal was anesthetized and perfused with the same fixative as above (2% paraformaldehyde and 2.5% glutaraldehyde in a 0.1 M sodium cacodylate buffer (pH 7.4)), and the brain was coronally sliced at 100 *μ*m thickness. The slices were processed with the original 3D-volume EM protocol by Deerinck et al. (2010), which is also listed as protocol 1 in Table 1.

### SBF-SEM Image Acquisition and Volume Data Handling

Tissue was sectioned and imaged using 3View2XP and Digital Micrograph (ver. 3.30.1909.0, Gatan Microscopy Suite) installed on a Gemini SEM300 (Carl Zeiss Microscopy LLC.) equipped with an OnPoint BSE detector (Gatan, Inc.). The detector magnification was calibrated within SmartSEM imaging software (ver. 6.0, Carl Zeiss Microscopy LLC.) and Digital Micrograph with a 500 nm cross line grating standard. A low-magnification image of each block-face was manually matched to its corresponding depth in the CLSM z-stack in Photoshop using blood vessels and cell bodies as fiducials. These features were clear across magnification scales (from 10× to ∼10,000×) and were used to estimate the *XY* position and depth of the cell and proximal segments of basal dendrites. Initial block-face correlation required several minutes to find the corresponding CLSM slice and overlay the two. Final imaging was performed at 2.0–2.2 kV accelerating voltage, 20 or 30 *μ*m aperture, working distance of ∼5 mm, 0.5–1.2 *μ*s pixel dwell time, 5.7–7 nm per pixel, knife speed of 0.1 mm/s with oscillation, and 56–84 nm section thickness. Acquisition was automated and ranged from several days to several weeks depending on the size of the ROI and imaging conditions. During acquisition, EM images taken from several depths were matched to CLSM z-stack images and were used to calculate a regression and predict the location of the cell in depth. This correlation was performed over the course of several days of SBF-SEM imaging. Pixel resolutions for each image volume were again calibrated following imaging with a 500 nm cross line grating standard using the same applied accelerating voltage and detector conditions used during imaging. Additionally, the true section thickness was measured using mitochondria diameter calibrations (Fiala & Harris, 2001). Calibration for each block was required, since variation in thickness can occur due to heating, charging from the electron beam, resin polymerization, and tissue fixation and staining quality (Starborg et al., 2013; Hughes et al., 2014).

Serial tiled images were exported as TIFFs to TrakEM2 (Schindelin et al., 2012) within ImageJ (ver. 1.52p) to montage tiles, then aligned using Scale-Invariant Feature Transform image alignment with linear feature correspondences and rigid transformation (Lowe, 2004). The tiling and alignment step required several days to complete on a dedicated workstation. Once aligned, images were inverted and contrast normalized. Each dendrite of interest was manually cropped from the full volume to reduce computational overhead in subsequent analyses. Aligned images were exported to Microscopy Image Browser (ver. 2.51, 2.6; Belevich et al., 2016) for segmentation of dendrites, spines, postsynaptic densities (PSDs), and boutons. Three annotators preformed segmentation on this project and the segmentations of each annotator were proof-read by an experienced annotator (∼1000 h of segmentation experience) prior to quantification. Binary labels files were imported to Amira (ver. 6.7, 2019.1, Thermo Fisher Scientific) which was used to create 3D surface models of each dendrite, spine, PSD, and bouton. Once reconstructed, the model of each dendrite was manually overlaid onto its corresponding 2-photon image using Adobe Photoshop for re-identification of individual spines. This manual correlation could be done in a matter of minutes. Amira was used to measure the volume of spine heads and boutons, surface area of PSDs, and spine neck length, which required several days of manual data analysis for each dendrite. Blender (ver. 2.79, 2.8) was used to create 3D renderings.

### Virtual Reality-Based 3D Data Correlation

We used the virtual reality software syGlass (ver. 1.4.0, IstoVisio) to correlate datasets. We began by opening both the CLSM stack and single block-face SEM image in syGlass, and thresholded the CLSM stack to reveal prominent blood vessels. Knowing which side of the tissue the block-face image was captured on, we used the slicing tool to crop to the relevant side of the CLSM stack, then used the “transformation” tool to apply a set of points manually to both datasets where prominent blood vessels could be visually identified and matched between them. Four matched points selected across each dataset were sufficient to successfully register the two. Correlation in VR could be completed within minutes.

## Results

### *Overview of Workflow to Correlate* In Vivo *2-Photon Imaging and SBF-SEM*

**Fig. 1.**
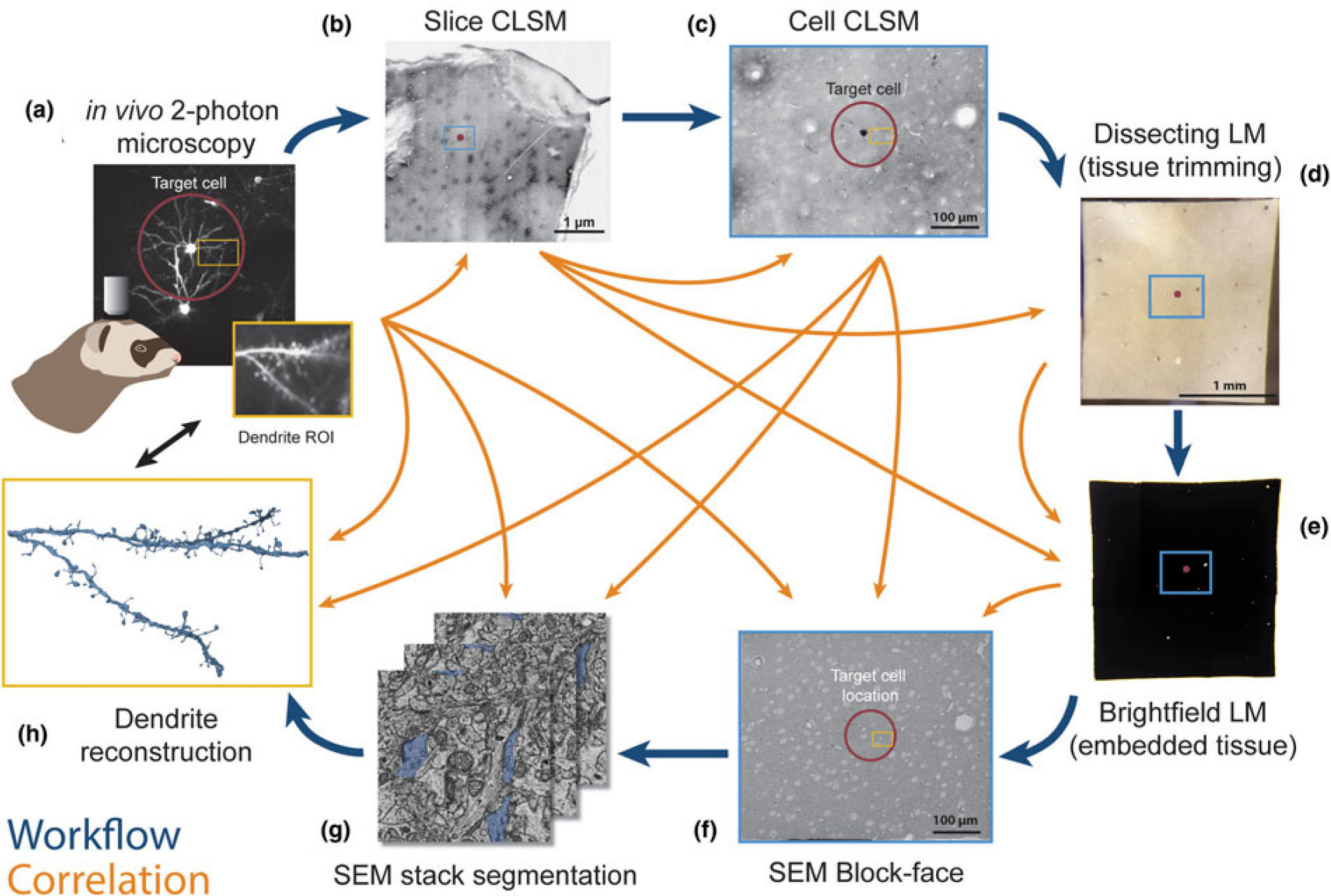
Workflow and correlative design for re-identification of cells from 2-photon live imaging to SBF-SEM. Blue arrows indicate the main workflow, while orange arrows indicate the direction of correlation. Red circles mark the target cell body and its position in the tissue, light blue rectangles demarcate a field of view containing the cell and landmark blood vessels throughout the workflow, and yellow rectangles demarcate the location of a dendrite of interest. (**a**) Dendritic spines of pyramidal neurons expressing GCaMP6s within the ferret visual cortex were functionally characterized *in vivo* using 2-photon LM. (**b**) Low-magnification CLSM image taken to locate the target cell in the slice relative to large blood vessels. (**c**) A higher-magnification CLSM z-stack image of the boxed area in (**b**), where the cell, its dendrites, nuclei of nearby cells, and capillaries are visible. (**d**) The slice was trimmed to a smaller size (<2 mm in width) for SEM sample preparation. (**e**) Following sample preparation, the stained tissue became opaque. (**f**) A block-face SEM image of the sample on a pin was correlated back to the 2-photon and CLSM image stacks and the desired imaging location was determined. (**g**) Serial SEM images were used for segmentation of the cell and its dendrites. (**h**) Target dendrites were reconstructed, morphologically characterized, and paired back to the initial functional data in (**a**).

**Fig. 2.**
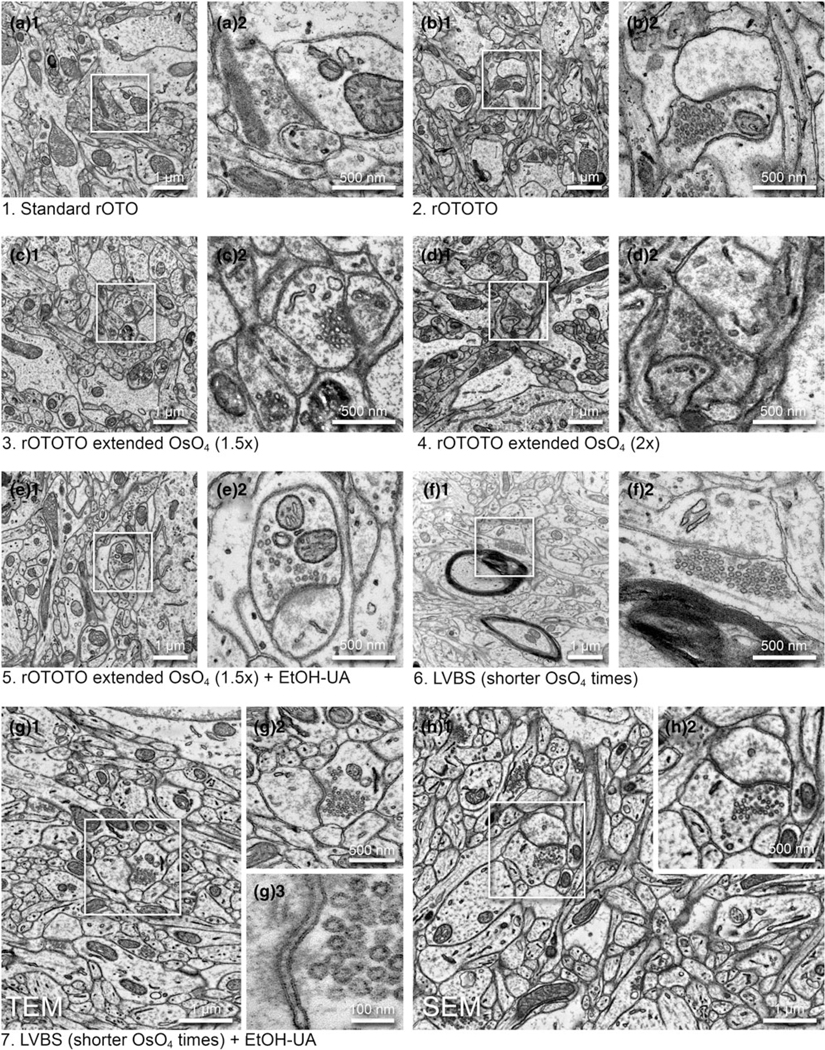
Optimization of a high contrast staining protocol for the ferret visual cortex. Representative images examined by TEM (**a–g**) and SEM (**h**) (1, medium magnification and 2, high magnification of the boxed area in 1) from the different protocols shown in Table 1. (**a**) The original rOTO protocol using reduced OsO_4_, TCH, and OsO_4_ before uranyl acetate (UA) and lead staining. (**b**) rOTOTO protocol, with tannic acid staining added before TCH. (**c**) rOTOTO plus elongated (1.5 times duration) osmium treatment. (**d**) rOTOTO plus elongated (two times duration) osmium treatment. (**e**) rOTOTO plus elongated (two times duration) osmium treatment and added UA-EtOH. (**f**) Modified LVBS method with shorter OsO_4_ staining. (**g**) Modified LVBS with shorter OsO_4_ staining plus UA-EtOH. Note, lipid bilayer of membranes was visible in this protocol (**g**_**3**_). (**h**) The block-face of the same sample as (**g**) was viewed using SEM.

**Fig. 3.**
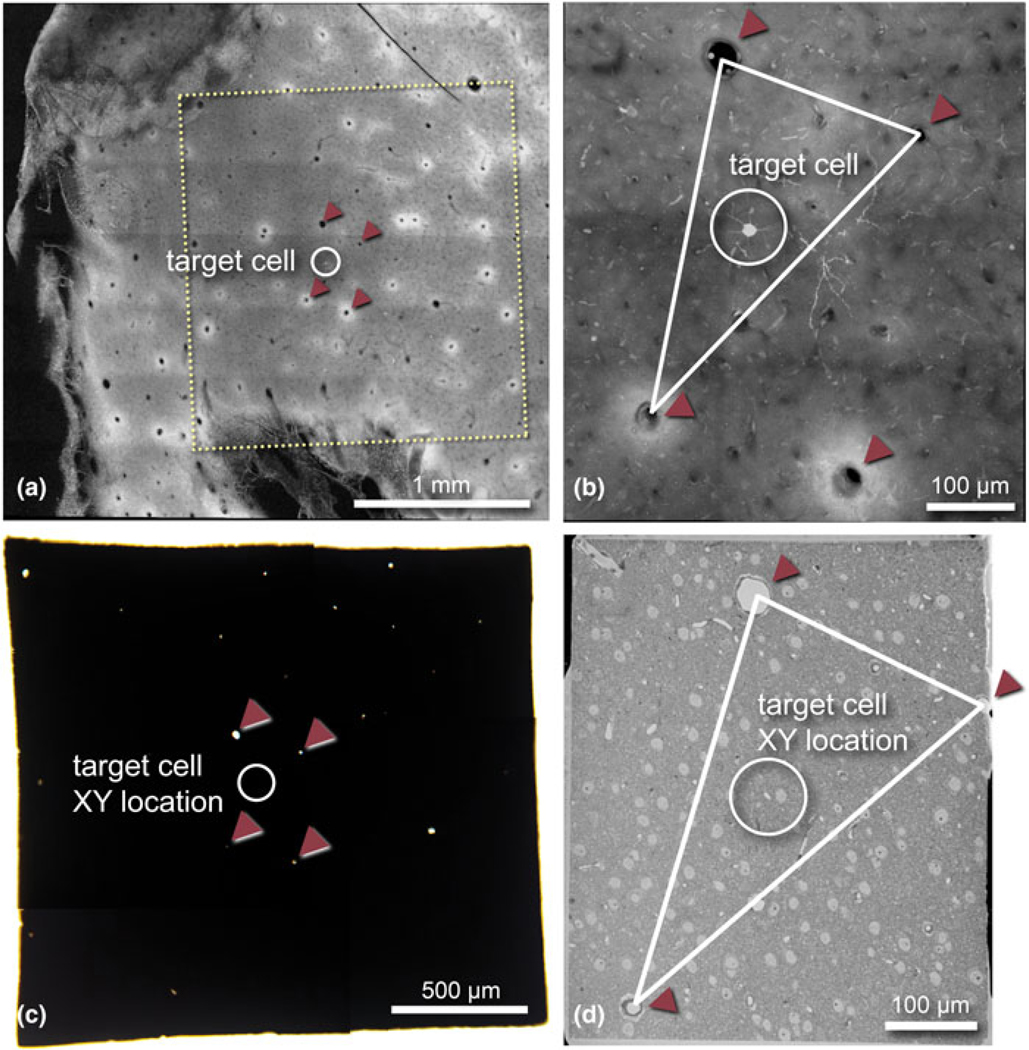
Blood vessels can be used as intrinsic fiducial markers to correlate image data collected across imaging platforms. (**a**) Low-magnification CLSM fluorescence image of a fixed cortical slice containing a target cell of interest (location marked with white circles). Blood vessels in this image can be easily identified as black circles. Note, glutaraldehyde autofluorescence manifests as white rings around vessels. Yellow box indicates the area trimmed for sample preparation as shown in (**c**). Red arrowheads point to four blood vessels which were key landmarks throughout the workflow. (**b**) Higher-magnification CLSM standard deviation projection image of the target cell and nearby vessels. Red arrowheads show the same blood vessels as in (**a**). (**c**) Bright-field LM image of the same sample embedded in resin. Note, only vessels which pass straight through the tissue can be visualized as bright points. (**d**) Block-face BSE image of the sample with three of the four landmark vessels visible. These were used to coarsely triangulate the location of the target cell using the CLSM image (**b**) as a reference (cell not yet exposed).

We established a correlative workflow from 2-photon *in vivo* calcium imaging to SBF-SEM to investigate ultrastructural characteristics of functionally identified synapses (Fig. 1). Functional imaging of dendritic spines was performed with *in vivo* 2-photon imaging (Fig. 1a) to characterize visually driven calcium activity of single cortical pyramidal neurons [see Scholl et al. (2020) for more details]. After fixation, we sliced the brain parallel to the indention of the cranial window to ensure subsequent imaging was performed in the same plane as the 2-photon microscopy images. Immediately following slicing, slices were imaged using CLSM to obtain low- and high-magnification images of the tissue (Figs. 1b, 1c). We trimmed each slice to a smaller square, placing the target cell in the center using blood vessels as fiducial markers (Figs. 1d, 3), then performed heavy metal staining with our optimized protocol (Figs. 1e, 2 and Table 1). We imaged the block surface with SEM for correlation to the CLSM image volume (Figs. 1f, 7). We then captured serial images of the area of interest with SBF-SEM (Fig. 1g) and performed segmentation and 3D reconstruction of the target spines on the dendrites (Figs. 1h, 8). Details of the individual steps in this workflow are described in the following paragraphs.

### Optimization of the Sample Preparation Method for SBF-SEM with Ferret Cortex Tissues

We tested several protocols to achieve uniform staining, high conductivity, and good cytoplasm-membrane contrast in SBF-SEM imaging to visualize the synaptic structure in ferret visual cortex samples. The original “SBEM” protocol (Deerinck et al., 2010) was used as a baseline and was modified by adding or changing reagents and adjusting durations (Table 1). The sample prepared with the original protocol (rOTO, protocol 1 in Table 1), which stained samples with reduced osmium tetroxide (OsO_4_), thiocarbohydrazide (TCH), and OsO_4_ before uranyl acetate (UA) and then lead, generally had good contrast (Fig. 2a). As expected, we could observe somata, myelin, neuropil, and synapses containing synaptic vesicles (SVs) with distinct membranes and slightly electron-dense cytoplasm (i.e., not “washed-out”; Fig. 2a_1_). However, the relative contrast for PSDs was weak and sometimes it was difficult to see excitatory synapses (Figs. 2a_2_). To “accentuate” the organelle-membrane contrast, we added a step with a tannic acid (Persi & Burnham, 1981) after the first reduced osmium treatment (Fig. 2b and Table 1, protocol 2), and further elongated the durations of osmium treatments (Figs. 2c, 2d and Table 1, protocols 3 and 4). In these protocols, the electron density of the whole tissue was increased as shown in low-magnification views (Figs. 2b_1_, 2c_1_, 2d_1_), and both membranes and cytoplasmic contents, including PSDs, became darker (Figs. 2b_2_, 2c_2_, 2d_2_). In the samples treated with longer OsO_4_ exposure, we observed small voids in mitochondria and synaptic vesicles, likely caused by insufficient resin infiltration (Figs. 2c_2_, 2d_2_). For the purpose of 3D reconstruction, high contrast of membranes relative to cytoplasm is helpful to demarcate and follow neurite profiles through sections. To that end, we tested the application of UA dissolved in ethanol (UA-EtOH) which reduced overall cytoplasmic staining intensity while maintaining electron density at the PSD (Fig. 2e). Despite this improvement, samples had inhomogeneous staining, where tissue further from the edge of the block tended to be poorly stained. To overcome this, we applied a large volume block staining (LVBS) method which allows OsO_4_ solution to infiltrate into the tissue before being reduced by potassium ferrocyanide to ensure even staining throughout large sample blocks (Hua et al., 2015). The overall staining became homogeneous (Fig. 2f and Table 1, protocol 6), but we observed less intense staining, likely because we reduced osmium incubation times to account for the thinner tissue slices used in our experiments than the reported samples. Our final test was to incorporate a UA-EtOH step to the LVBS protocol 6 (Table 1, protocol 7). This protocol resulted in homogeneous staining throughout the sample and good membrane to cytoplasm contrast, with visible PSDs in our ferret visual cortex samples (Fig. 2g), and we selected this protocol for subsequent experiments. We also note that this protocol provided clear visualization of the lipid bilayer under TEM observation (Fig. 2g_3_, inset). Following TEM characterization, we tested image quality of the sample prepared with protocol 7 using SBF-SEM (Fig. 2h). We observed exceptional membrane preservation, cytoplasm-membrane contrast, and the neuropil could be imaged with a high enough quality for segmentation at a 2 kV acceleration voltage and relatively fast dwell time of 2 *μ*s at a current of 1 nA without charging, which indicated the sample had a sufficient electron conductivity. The PSD was clearly visible with SEM imaging using this protocol (Figs. 2h_2_).

### Correlative Specimen Trimming for SBF-SEM Using Biological Fiducial Markers

For correlative experiments, we imaged tissue slices using CLSM to obtain 20× magnification tiled images of the slice (Fig. 3a). This allowed us to identify our fluorescent target cell as well as nearby large blood vessels for fiducial markers. In order to capture smaller capillaries and obtain a 3D representation of the tissue, we captured a higher-magnification z-stack of the area with the target cell (Fig. 3b). Using this as a guide, we trimmed the slice from 1.5 × 1 cm to a smaller 1–2 mm^2^ with the target cell near the center, and performed sample preparation for SBF-SEM (Fig. 3c). Trimming was done to achieve more homogeneous staining, better resin infiltration, keep slices flat during sample preparation, and leave only a few key landmark blood vessels to reduce confusion during future correlative steps. After heavy metal staining, tissue became fully black and opaque, and often only approximately a dozen vessels which pass vertically through the tissue depth could be seen under a dissecting or bright-field microscope (Fig. 3c). In 100% of slices we observed, there were enough of these vessels to perform correlation. We used these vessels as landmarks to correlate to the low-magnification CLSM image (Figs. 3a, 3c, red arrows) and identify the cell’s approximate location (Fig. 3a). The arborization of targeted dendritic ROIs spanned no more than 300 *μ*m across; therefore, using the information of the cell’s location, we were able to trim each sample to a height and width of approximately 800 *μ*m. Upon mounting to an aluminum pin with silver epoxy, any large vessels remaining in the block after trimming were visible under the dissecting microscope. The exposed surface of the block-face was then imaged at low magnification on the SEM (Fig. 3d), which provided a complete view of vessels and somata at the surface and was used to correlatively triangulate the *XY* position of the target cell with the fluorescence view (Fig. 3b).

### Virtual Reality Assisted Block-Face Correlation Using Blood Vessels as Fiducial Markers

**Fig. 4.**
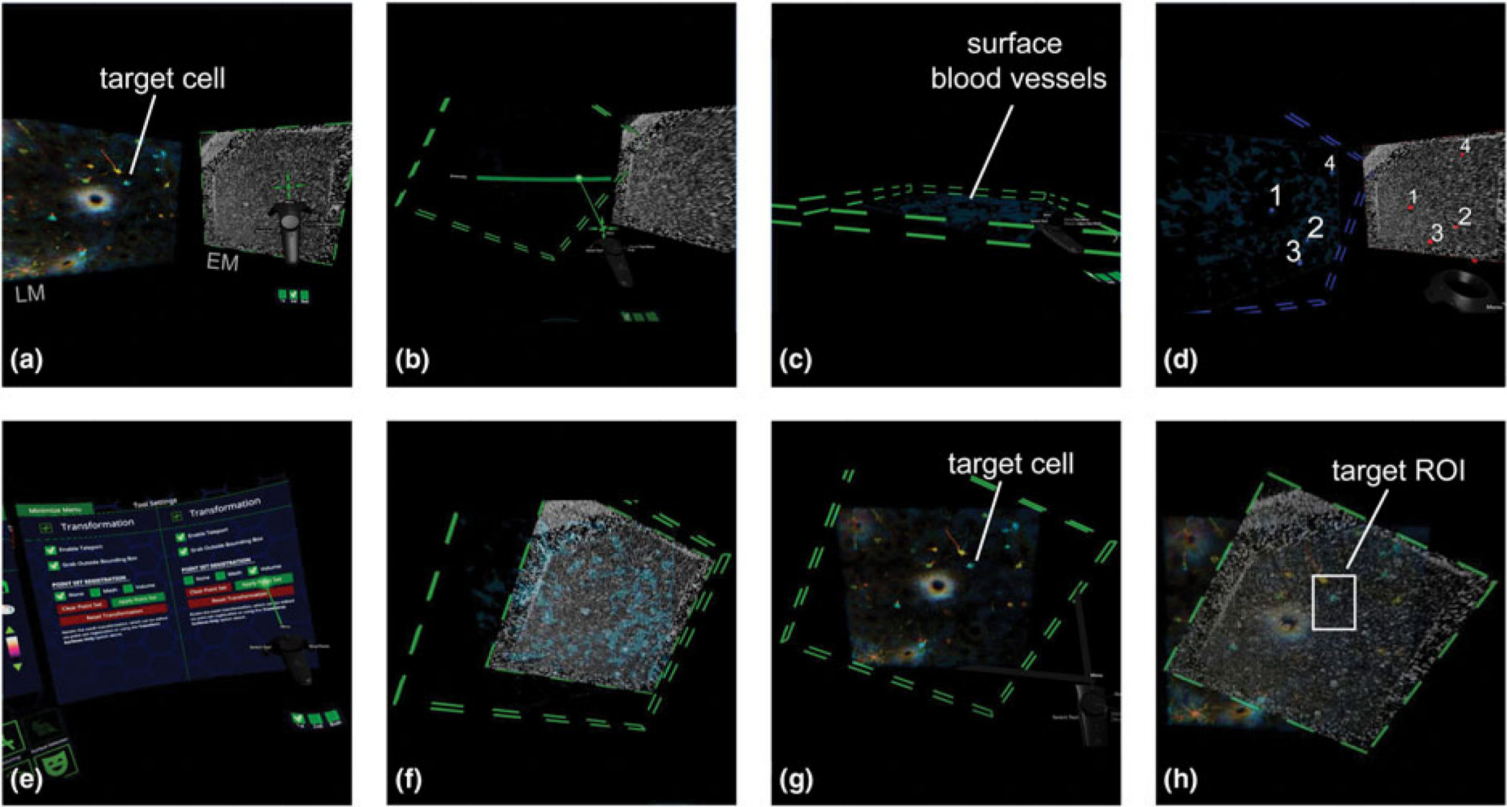
Virtual reality assisted correlation allows for precise estimation of cell coordinates. (**a**) The CLSM and EM datasets were loaded into syGlass, and the target cell was located within the CLSM image data. (**b**) Thresholding of the LM displays blood vessels. (**c**) Surface blood vessels were selectively visualized using the slicing tool. (**d**) The transformation tool was used to place markers on matching blood vessels in both datasets. (**e**) Point set correlation was applied to automatically match the EM to LM. (**f**) View of the correlation results. Note, the blood vessels can be viewed through the correlated EM dataset. (**g**) With the slicing tool, the EM image can be hidden and the CLSM image can be reset to show cells. (**h**) Changing the EM image opacity allows the user to determine a target ROI for imaging (coordinates on the EM image can be exported as a .csv file).

To achieve a precise estimation of the cell coordinates relative to the blood vessels that were visualized by CLSM, we applied a newly developed registration tool in virtual reality (Fig. 4; Supplementary Movie 1). This could be done as an alternative to a more conventional method of identifying the cell position in a stack of images on a computer monitor, which required back-and-forth checking of serial images many times. We imported both the single exposed block-face image as well as the CLSM volume z-stack data of the tissue into the virtual reality software syGlass (IstoVisio), which allowed us to align the single block-face EM image to the CLSM z-stack data in 3D space. By first thresholding the CLSM stack to only display blood vessels, fiducial markers could be easily placed to register the two datasets. Once registered, we were able to identify the expected location of the cell within the tissue (Fig. 4; Supplementary Movie 1). We used this information to then estimate the SBF-SEM imaging ROI required to capture the dendrites of interest from the target cell (Fig. 4h).

### Technical Advancements in SBF-SEM Provide Greater SNR and Reduce Sample Charging

**Fig. 5.**
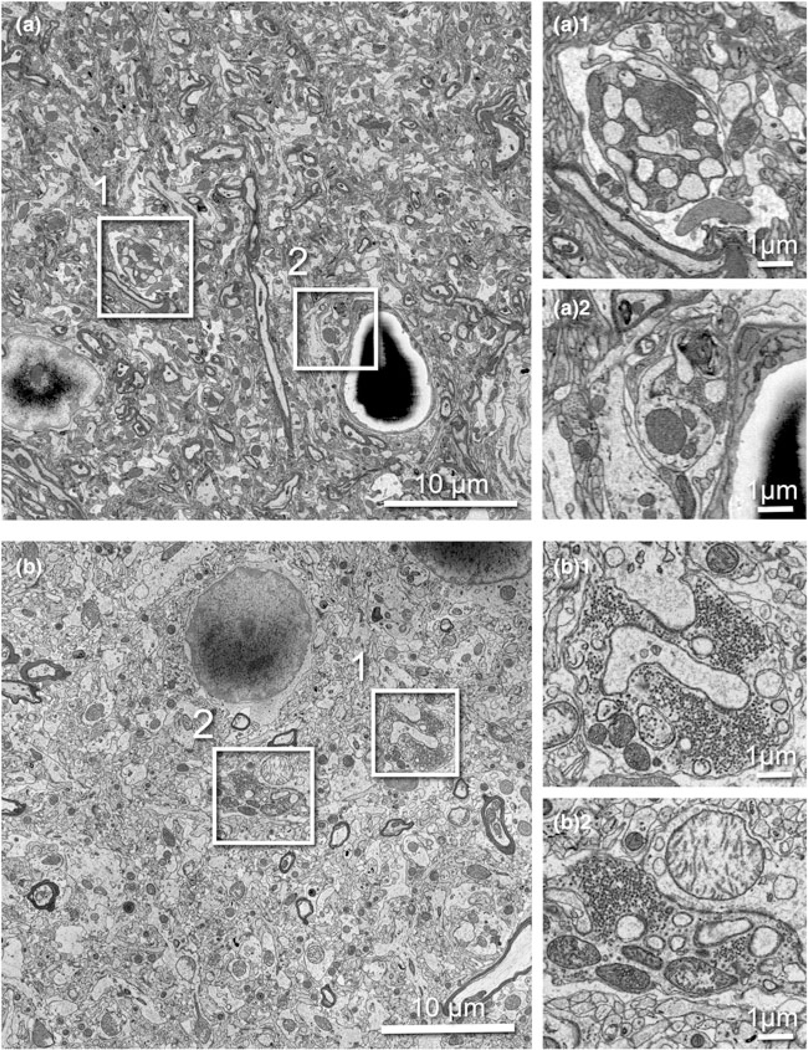
Comparison of serial 3View imaging of rat cortex captured using the original BSD versus OnPoint detector. Imaging conditions were optimized to obtain thinnest possible slices with highest SNR. (**a**) Representative image captured with the original 3View BSD (10 nm/pixel resolution and 1.0 *μ*s dwell). (**a**_**1**_ and **a**_**2**_) Two magnified regions showing a presynaptic terminal containing synaptic vesicles. (**b**) Representative image captured with the onPoint detector (8.2 nm/pixel resolution and 0.5 *μ*s dwell). (**b**_**1**_**,b**_**2**_) Two magnified regions showing a presynaptic terminal containing synaptic vesicles. Note individual vesicles are clearly visible, as are mitochondrial cristae. All boxed areas are three times digitally enlarged.

SBF-SEM imaging is challenging for non-conductive biological tissues. To obtain high SNR, sufficiently high electron dose is necessary; however, if the dose becomes too high, charging can cause image distortion, heating, and sample damage. We replaced the 3View backscattered-electron detector (BSD) with the OnPoint BSD (Gatan) and used it for correlative SBF-SEM imaging. The OnPoint detector was developed to have higher sensitivity to backscattered electron signal and faster imaging speeds in low accelerating voltage regimes (<5 kV) (https://www.gatan.com/products/SEM-imaging-spectroscopy/onpoint-bse-detector#-body). To compare the original 3View detector and the OnPoint detector, we tested the imaging conditions that enabled the thinnest slice thickness, smallest pixel resolution, and best SNR during the serial imaging process (Fig. 5). For the comparison test, we used a sample from rat cortex prepared with the standard rOTO protocol (as shown in Table 1, protocol 1) and used the same sample pin with both detectors. With the original BSD, we could serially cut and image the sample as thin as 70 nm at 2.2 kV, 10 nm/pixel resolution, and 1.0 *μ*s dwell time (Fig. 5a). With this detector, mitochondria cristae could not be resolved and synaptic vesicles in axon terminals were visible as a group, but not individually (Figs. 5a_1_, 5a_2_). When we examined the same pin with the OnPoint detector, we could section much thinner, 45 nm, for serial imaging using 1.4 kV acceleration voltage, 8.2 nm/pixel, and 0.5 *μ*s dwell time (Fig. 5b). We observed an increased SNR with this detector and organelles and membranes appeared much crisper. Specifically, mitochondria cristae could be visualized and individual synaptic vesicles in terminals could be clearly identified and counted (Figs. 5b_1_, 5b_2_).

**Fig. 6.**
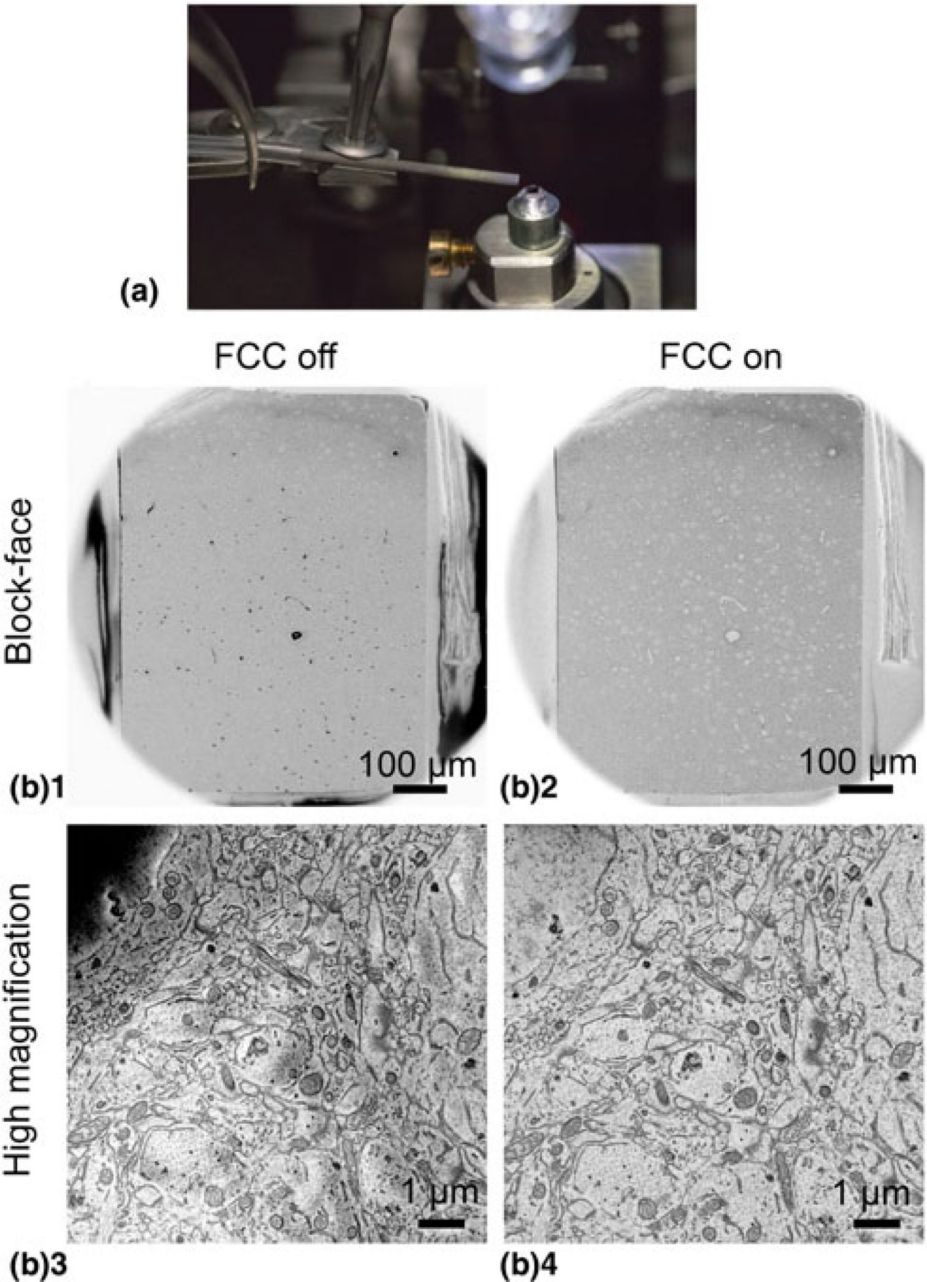
FCC reduced charging in samples lacking conductivity. (**a**) The FCC module equipped on a gatan 3View system. A carbon fiber needle delivers nitrogen gas to the block-face and can be adjusted with a set screw. (**b**_**1**_) Low-magnification block-face SEM image of low conductivity tissue without FCC activated. Notice severe charging (black) in areas composed mostly of resin (e.g., blood vessels and cell somata). (**b**_**2**_) The same view with FCC activated. (**b**_**3**_) High-magnification image of the same tissue without FCC activated. Without FCC, charging is severe in the nucleus (top left) and produces a “shadow” effect in neurites. (**b**_**4**_) The same view with FCC activated.

Furthermore, we applied the focal charge compensation device (FCC, Zeiss) to reduce charging artifacts created by a lack of sample conductivity (Fig. 6). With this device, nitrogen gas is released through a carbon fiber needle onto the surface of the block-face (Fig. 6a; Deerinck et al., 2018). Secondary electrons leaving the surface of the sample ionize the nitrogen gas, which then neutralize negative charge on the sample surface. Without charge compensation, we observed severe charging in blood vessels and nuclei, areas mostly composed of empty resin, particularly in samples previously imaged with LM (Fig. 6b_1_). Charging in the neuropil caused a “shadowing” effect and produced a distorted image compared to the image of the same area with charge compensation applied (Fig. 6b_3_). We found that a gas injection setting of 60% within the software was sufficient to completely remove charging artifacts at both moderate and high magnifications; however, settings higher than 40% tended to create unstable vacuum conditions over time within the microscope and did not permit long duration imaging sessions (i.e., several weeks). Because of this, we used 30–40% gas injection for long runs, which was sufficient to reduce almost all charging (Figs. 6b_2_, 6b_4_), and closely monitored the SEM vacuum status throughout the imaging duration.

### Identification of the Target Cell in EM Data Using Depth Correlation

**Fig. 7.**
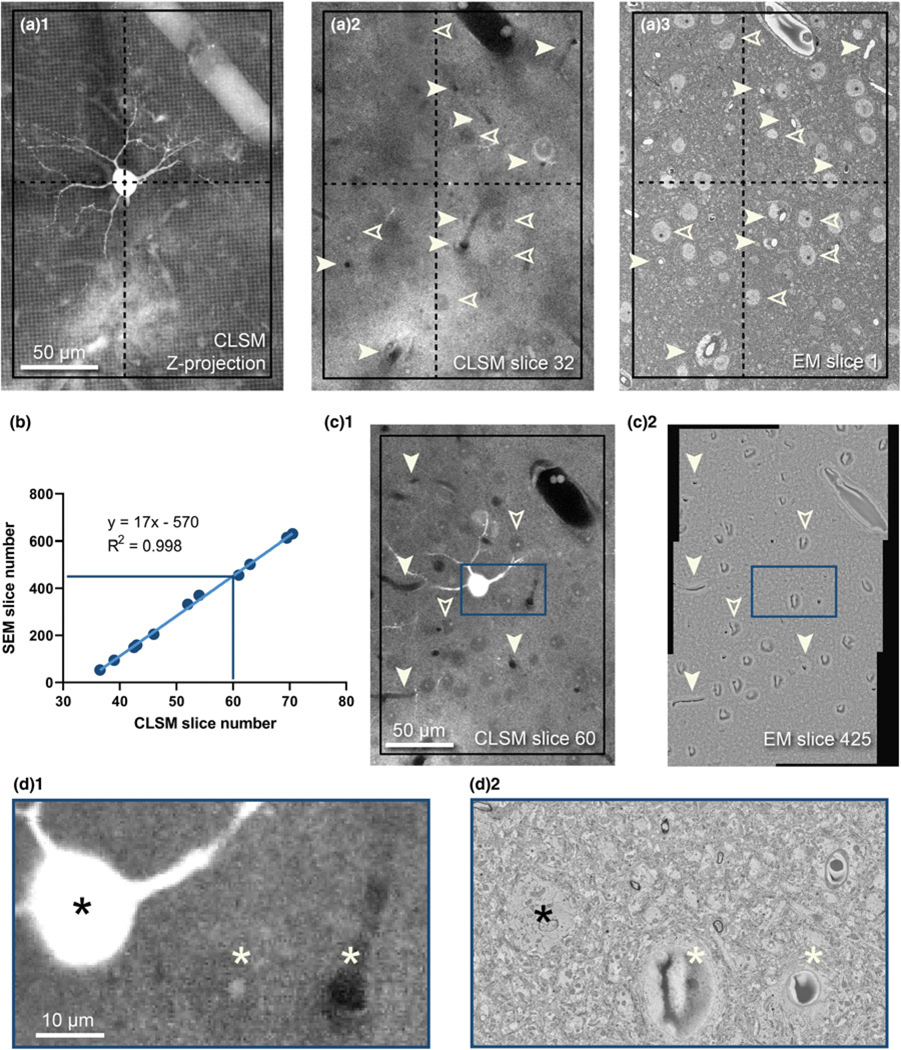
Depth correlation of the target cell. (**a**) Fine *XY* correlation beginning at the surface of the sample. (**a**_**1**_) *Z*-projection of a target cell and its basal dendrites from the CLSM stack, framed within a prospective imaging ROI (black box) with the cell soma marked by a crosshair. (**a**_**2**_) A single slice selected from the same CLSM stack to match with the first SEM block-face image before sectioning started (**a**_**3**_). Paired blood vessels (solid arrowheads) and cell somata (outlined arrowheads) can be identified between both images. (**b**) Regression showing the *Z*-depth of the target soma in an SEM dataset can be estimated from its depth in the CLSM stack. (**c**_**1**_) The soma is visible in slice 60 of the CLSM stack, and as estimated by the regression, also appears in slice 425 of the EM stack (**c**_**2**_) along with other landmarks (arrowheads). The EM image shown is tiled, stitched, contrast normalized, and down-sampled to 3% of the size of the raw data. (**d**) Zoomed areas of the boxes in (**c**) showing the target cell (black asterisk), as well as two nearby identifying features—a nucleus and a capillary (white asterisks) visible by CLSM (**d**_**1**_) and EM (**d**_**2**_).

For volume imaging, we took a targeted approach to image only to the depth required for capturing all target dendritic spines. This was done to minimize imaging time and computational load and was achieved by continuous correlation of features between the CLSM z-stack and captured EM images (Fig. 7). To begin, the standard deviation projection of the CLSM stack was used to identify the position of the cell soma in the tissue and determine the size of the ROI necessary to capture the target dendrites (Fig. 7a_1_, black rectangle). A single image from the stack was identified which matched the appearance of the initial block-face EM image (Figs. 7a_2_, 7a_3_)—using blood vessels and cell nuclei as correlative markers (see Fig. 3) and observation of the images in virtual reality (see Fig. 4). We used this information to determine the size of the imaging field of view and to set up pixel resolution and image tiling for the SEM (Fig. 7a). We used the montage function of Digital Micrograph to capture 2 × 2 or 2 × 3 rectangular tiles of between 10,000 and 17,000 pixels in either dimension. This was enough to cover the dendritic field proximal to the cell soma that was functionally imaged by 2-photon LM. While EM imaging and sectioning were underway, tiled images were periodically retrieved from the output folder and stitched together. These images were compared to the CLSM image stack, and the corresponding CLSM slice was identified using inherent fiducials. At the end of serial imaging, we performed a regression between EM section number and corresponding CLSM slice number (Fig. 7b), which we then used to estimate the depth and identify the target soma within the EM image data (Figs. 7c, 7d).

### Correlation and Segmentation of Target Dendrites and Dendritic Spines

**Fig. 8.**
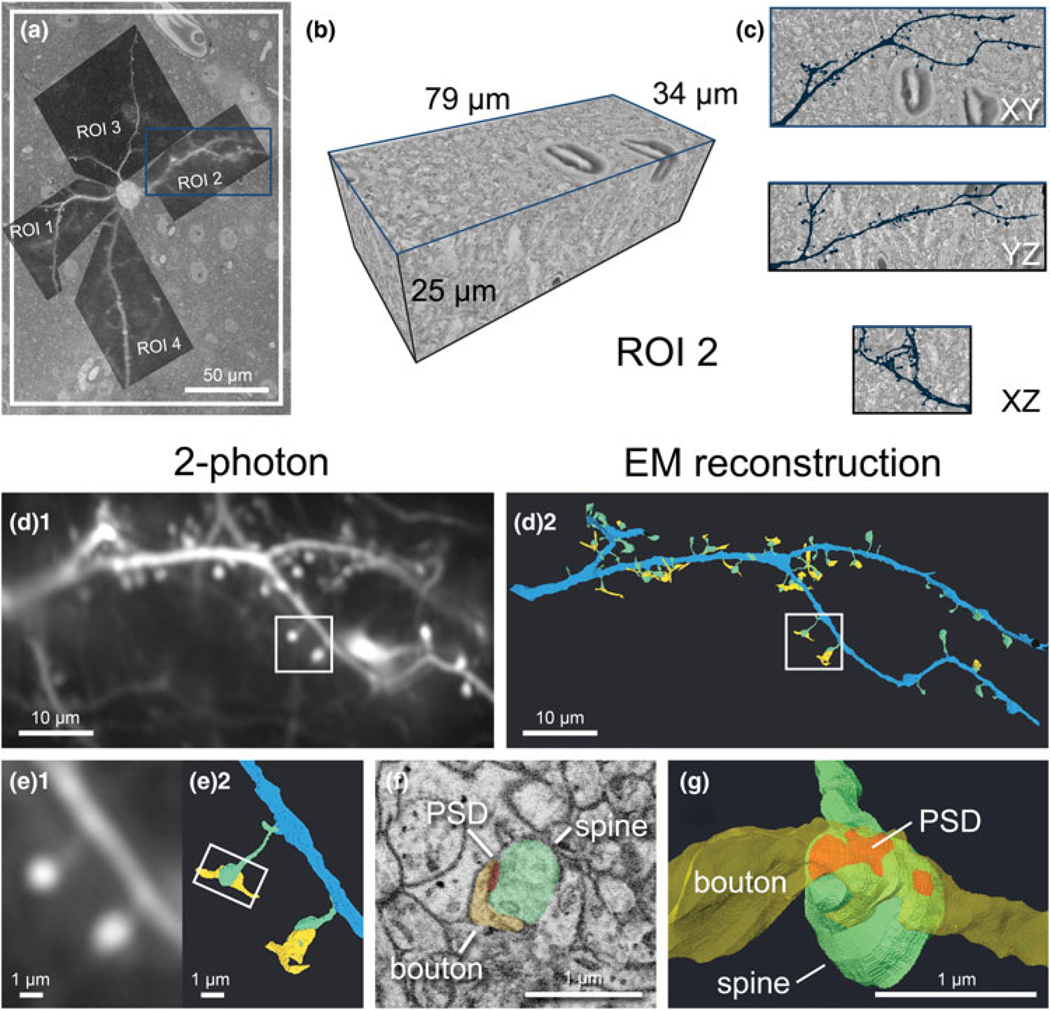
Cropping of dendritic ROIs spine identification and image analysis. (**a**) 2-photon LM image of dendritic ROIs which served as a template for cropping. (**b**) An example volume of 3D rendered EM data containing the dendritic segment of ROI 2 corresponding to the blue box in (**a**), identified by following it outwards from the soma. (**c**) Orthogonal views of the same volume, with the EM reconstruction of the dendritic segment overlaid in dark blue. (**d**) The final comparison of the initial 2-photon image (**d**_**1**_) and 3D EM reconstruction of the same dendrite (**d**_**2**_). The dendrite is segmented in blue, spines in green, portions of axonal bouton in yellow, and PSD in red (for panels **d–g**). (**e**) Magnified view of the spines boxed in (d) viewed by 2-photon LM (**e**_**1**_) and 3D reconstructed EM (**e**_**2**_). (**f**) Representative SEM image showing the ultrastructure of synapse boxed in (**e**). (**g**) Magnified 3D EM reconstruction of the same spine showing the different components of a synaptic contact.

Following acquisition, all images were processed in TrakEM2 to montage tiles, align z-sections, invert, and normalize contrast. After processing, the acquired SEM image stacks were ∼2–4 TB per target cell. Therefore, we cropped multiple smaller volumes containing dendrite(s) of interest from the dataset for segmentation using MIB. By overlaying the 2-photon images of each dendrite on the top of the SEM image containing the soma (Fig. 8a), we could identify each dendrite and follow them outwards from the soma through the EM dataset within TrakEM2. Dendritic branch points, visually prominent spines, and small capillaries near the dendrite were used to determine size of the volume needed to encompass all spines of interest, and the cropped datasets ranged from 4 to 47 GB in size (Fig. 8b). We used manual segmentation to annotate and reconstruct target dendrites, dendritic spines, PSDs, and axonal boutons, which could be visualized within the cropped volume (see ROI 2 as an example, Fig. 8c). We matched reconstructions to their corresponding 2-photon image to spatially correlate and identify spines of interest (Figs. 8d, 8e), then morphological measurements were made (Figs. 8f, 8g) and correlated to their functional properties. Our workflow allowed for reliable data collection and the number of function-paired spines analyzed was about ten times higher than previous reports [see Scholl et al. (2020) for numerical results].

## Discussion

We developed a CLEM workflow centered on utilizing biological fiducial markers to identify target neurons across microscopy modalities without the use of EM labels. This strategy allowed us to pair functional properties with ultrastructural characteristics of synapses in ferret visual cortex. The workflow was optimized to provide high reproducibility and consistency to obtain a large number of reconstructed synapses with sufficient resolution to analyze synaptic architecture using SBF-SEM, and to correlate that back to the initial 2-photon *in vivo* functional imaging data. With this paired data, we were able to statistically analyze and discuss the basis of synaptic strength in a brain network (Scholl et al., 2020).

### Intrinsic Biological Features Were Sufficient and Non-Disruptive Correlative Fiducial Markers for Identifying Pyramidal Neurons in Ferret Visual Cortex

Using morphological features such as blood vessels and somata as correlative markers has several advantages over EM labeling methods which use an immuno-gold or peroxidase/DAB reaction to identify target cells (Goetz, 2018). The most significant benefit is excellent preservation of the ultrastructure of the tissue, as it can be fixed with a higher concentration of glutaraldehyde (up to 2.5%) than what is common with immuno-gold labeling (<0.5%) (Polishchuk & Polishchuk, 2019). We found that the green fluorescence signal of GCaMP6 in pyramidal neurons was strong enough to permit their CLSM imaging following strong glutaraldehyde fixation, despite the presence of autofluorescence in the tissue. In fact, the strong glutaraldehyde autofluorescence we observed immediately surrounding blood vessels conveniently assisted thresholding of vessels from the CLSM z-stack for correlation. Other benefits of using inherent fiducials are that blood vessels are visible throughout the sample preparation process, they range orders of magnitude in diameter, and often weave complex paths through the tissue. This makes them ideal fiducials for *X*, *Y*, and *Z* correlation at any step of the workflow, from dissecting scope to SEM. Nuclei are the other features that stood out due to glutaraldehyde autofluorescence. Specifically, cell nucleoli had bright signal on a darker background of the nucleoplasm in CLSM images and were used during correlation at moderate magnifications as shown in Figure 8d. Yet another benefit of using inherent fiducials is that the time required for EM sample preparation is reduced significantly from upwards of a full week with the immuno-gold labeling techniques to 4 days. Much of this time is gained from abolishing several days of antibody incubations.

Although variability exists in the arrangement of blood vessels across slices, we were able to successfully perform landmark correlation in 100% of slices we prepared. Heavy metal staining turned tissues opaque and obstructed the view of any vessels which did not pass straight through the tissue. Nonetheless, a sufficient number of these vessels existed in the tissue to make landmark-based correlation possible following tissue embedding. It may be that the prolific vascularization of the cortex made this possible, and other brain regions or thicker tissues may be more difficult to correlate. In a case where vessels are not easily visible for correlation following EM sample preparation and embedding, we confirmed that collecting a semi-thick section from the entire piece of embedded tissue and staining it with toluidine blue to visualize the position of the larger vessels at the surface of the tissue could be done as an alternative. By correlating these, the tissue can be further trimmed to the desired size for SBF-SEM.

### Modification of Heavy Metal Treatments Provided Homogeneous Staining and Excellent Cytoplasm-Membrane Contrast

Intense heavy metal staining is essential for SBF-SEM as imaging of bulk samples *in situ* requires high conductivity (Tapia et al., 2012). Traditionally, this is achieved with multiple osmium incubations and enhancement steps involving potassium ferrocyanide, thiocarbohydrazide, tannic acid, which provide staining of lipids. Uranyl acetate and lead aspartate are also important contrasting and conductive agents, the latter of which has been shown to be crucial for conductivity (Genoud et al., 2018). Adapting previous work, we tested several protocols for optimal staining of 80 *μ*m thick slices of ferret visual cortex (Table 1). Many of the protocols we tested stained the cytoplasm contents and membranes too intensely, provided inhomogeneous staining through the tissue’s depth, or did not sufficiently contrast membranes from cytoplasm. We applied a method [termed as “large volume block staining (LVBS)” protocol; Hua et al., 2015] that separates OsO_4_ and potassium ferrocyanide incubations to improve staining consistency throughout the tissue. With this preparation, we observed homogeneous staining through the tissue’s depth but not well-contrasted staining of cytoplasm and membranes. We also incubated tissues in ethanolic UA for a short period of 20 min. Ethanol alone is known to form precipitate in the presence of phosphate buffer, but not cacodylate buffer, particularly at high ethanol concentrations, and 2% UA in 70% ethanol has been shown to cause precipitate in either buffer (Colquhoun & Rieder, 1980; Louw et al., 1990). We did not observe any evidence of precipitation, perhaps because we used a milder 1% UA dissolved in 25% ethanol. Ethanol is known to vastly accelerate the ability of UA to stain cytoplasmic constituents throughout the tissue; we saw extreme cytoplasmic staining intensity after just a 30-min incubation, while anything less than 15 min was too light in our series of tests (data not shown; Hayat, 1981). Our 20-min incubation was just long enough to add electron density to the cytoplasm and provide excellent contrast simultaneously, which helped the image analysis process. Having a lighter cytoplasm relative to membranes facilitated segmentation using semi-automated methods present in the segmentation software MIB such as watershed segmentation, which increased segmentation throughput. Our protocol also provided clear PSDs, which can be difficult to properly stain in SBF processed samples (Mikula et al., 2012). Combining both the LVBS protocol and ethanolic UA, we were able to consistently achieve homogeneous and high contrast staining for tissue slices from the ferret visual cortex.

### Virtual Reality Made Correlation More Intuitive by Directly Overlaying Two Data Sets in a 3D Space

SBF-SEM imaging by nature is destructive; the user has one opportunity to capture an area of interest, as sections cut from the block are discarded. Therefore, precise *XY* localization of the target cell and dendrites within the sample is key and allows the user to accurately set up an SEM imaging ROI, while the features are still embedded within the sample. We used virtual reality (VR) to approximate the target cell’s location relative to intrinsic landmarks within the slice using the CLSM volume and the initial block-face image of the sample. VR is a relatively new addition to the image analysis toolbox, and several companies have created software packages that allow users to analyze and process data in a 3D or 4D immersive space (arivis, VisionVR; IstoVisio, syGlass). The technique has largely been utilized for teaching and demonstrations rather than actually assisting in data collection or analysis; however, its use as a platform for combining structural and functional cellular data in a four-dimensional space and as a tool for simulation has been discussed (Parton, 2020). In many correlative projects, successful relocation of target features is driven by the researcher having a strong understanding of the characteristics of each dataset. We found that VR facilitated a more natural interaction with 3D data and helped the researcher become familiar with the morphological features present within the volume, which was useful when trimming the sample and for setting up volumetric EM imaging. For example, using VR for this purpose provided an advantage over simple 2D registration, as slight adjustments to the angle of the block-face image could be freely and easily manipulated with the controllers. The target cell’s depth within the tissue could also be measured within VR and made it easier to pick out the soma from the EM volume. The ease of use and intuitive interaction with the data in VR means correlation of the EM block-face to the CLSM data can be achieved in a comparable amount of time to manual 2D image overlay, and in most cases is even faster (2–3 min from loading the data to registration). Nonetheless, this step requires the user to have certain hardware, including a headset, controllers, and a workstation with above-average graphical capabilities.

### Image Quality Was Enhanced Using a High-Sensitivity Detector and FCC Module

Even when properly stained, resin-embedded brain tissue contains regions of low metal content such as blood vessel lumen and cell nuclei. Furthermore, samples which have been previously imaged with 2-photon and confocal microscopy sometimes show structural degradation, potentially from photodamage (Denk & Svoboda, 1997), which we observed can cause lower conductivity compared to samples not examined with LM (Figs. 3, 6b). Correlative experiments have a much lower success rate than pure EM morphological studies, and due to the high cost of per forming CLEM, it is unacceptable to reach the SBF-SEM imaging step only to find the sample is unusable because of charging. Therefore, in cases where charging of the neuropil was enough to disrupt imaging, we applied the FCC unit originally developed by Deerinck et al. (2018) and produced by Carl Zeiss, which delivers a stream of nitrogen gas at the sample surface to neutralize negative charge buildup. One parameter that directly affects charging is electron dose, which is heavily influenced by beam dwell time. Short dwell times are ideal to minimize charge buildup, but result in a lower SNR. Using the OnPoint detector from Gatan, we were able to image at dwell times as low as 0.5 *μ*s at low kV, while maintaining enough signal to identify synaptic features. This faster imaging allowed us to capture larger fields of view in reasonable time-frames, which was essential for imaging several ROIs. Using the original 3View BSD required longer dwell times and higher voltage to achieve a similar amount of SNR, but never reached the level of quality we saw with the OnPoint detector.

### The Precision of Our Approach Allows Efficient Structure-Function Mapping at the Scale of Individual Synapses

Our approach to correlation relied on first finding the target cell soma, as it is the largest and most obvious portion of the neuron and is identifiable at low magnifications. Because we sliced brains parallel to the cranial window used for 2-photon imaging, the imaging plane was nearly identical between confocal and electron microscopy, limiting sectioning-plane angle offset between datasets and making the correlation process more straightforward. We precisely estimated the distance that functionally imaged dendrites spread outward from the soma using correlation in Photoshop, VR, and TrackEM2 before beginning volume-EM imaging. This enabled us to maximize spine recovery rate while reducing the imaging size and time for each volume. Similar to work by Luckner et al. (2018), we show that individual spines can be identified using this precise correlation of inherent fiducials. Where they showed this could be done with FIB-SEM of minimally embedded brain slices, we show this is possible also with flat-embedded slices and large fields of view using SBF-SEM.

Experiments involving dense reconstruction are often several TB in size and benefit from software which can handle annotations on extremely large image data (Kornfeld et al., 2020). To capture dendritic arborization while imaging at synaptic resolution, our datasets also ranged from 2–4 TB in size. Nonetheless, our experiments with *in vivo* LM involved sparse labeling of neurons; therefore, the volume of the cell imaged by LM was much smaller than the total volume acquired by EM. Thus, to take advantage of features such as semi-automated segmentation which are present in MIB and increase segmentation throughput, we chose to extract sub-volumes of dendritic arbors for annotation. By precisely correlating data across multiple imaging platforms, we shortened the lengthy and computationally demanding image processing steps of this workflow, making this workflow faster and more accessible.

## Summary

We have established an efficient correlative workflow for targeting neurons and synaptic features with SBF-SEM that were previously imaged with 2-photon *in vivo* LM. This workflow uses inherent landmarks such as blood vessels and cell nuclei to triangulate the position of a target cell within a slice of tissue, from sample preparation to SEM imaging. Our goal was to establish a reliable and reproducible methodology to quantitatively characterize ultrastructure of synapses within large volume data sets using currently available software products and standard workstations. We could then pair anatomical data with functional properties to better understand neural circuit organization. In the future, further correlative investigations of brain networks of larger volumes in reasonable time-frames will require some breakthroughs in imaging and analysis techniques.

## Supplementary Material

Thomas et al Supplementary Materials

To view supplementary material for this article, please visit https://doi.org/10.1017/10.1017/S1431927620024757.

## Figures and Tables

**Table 1.** Different Protocols Tested for Optimization of Sample Preparation for the Ferret Visual Cortex.

	Protocol	Reduced OsO_4_		TA	OsO_4_	TCH	OsO_4_	UA	Lead Aspartate	Synaptic Vesicles	PSD	Cytoplasm Contrast	Membrane Contrast
1	rOTO	60′		−	−	20′	30′	O/N (H_2_O)	30′	+	+/−	+	
2	rOTOTO	30′		30′	30′	20′	30′	O/N (H_2_O)	30′	++	+	+	
3	rOTOTO (OsO_4_ ×1.5)	30′		30′	45′	20′	45′	O/N (H_2_O)	30′	+/−	+	+	
4	rOTOTO (OsO_4_ ×2)	30′		30′	60′	20′	60′	O/N (H_2_O)	30′	+	+/−	++	
5	rOTOTO (OsO_4_ ×2) + EtOH-UA	30′		30′	60′	20′	60′	20′ (20% EtOH)	30′	+	+	−	
		OsO_4_	Potassium ferrocyanide										
	LVBS	90′	90′			45′	90′	O/N (H_2_O) + 120′ at 50°C	120′				
6	Shortened LVBS	45′	45′			20′	45′	O/N (H_2_O)	30′	+	−	−	++
7	Shortened LVBS + EtOH-UA	45′	45′			20′	45′	20′ (20% EtOH)	30′	+	+	+	++
